# Supplementary material for: Genome sequences of two diploid wild relatives of cultivated sweetpotato reveal targets for genetic improvement
Source: Nat Commun. 2018 Nov 2;9:4580. doi: 10.1038/s41467-018-06983-8 (PMC6214957; doi:10.1038/s41467-018-06983-8)
Supplement: Supplementary file 1 — Supplementary Information [file 41467_2018_6983_MOESM1_ESM.docx]

## **Genome sequences of two diploid wild relatives of cultivated sweetpotato reveal targets for genetic improvement**

Wu *et al.*

## **Supplementary Method 1. Genome and transcriptome sequencing**

## **Plant materials**

The *I. trifida* line, NCNSP0306, which has a relatively low level of heterozygosity, and the highly homozygous *I. triloba* line, NCNSP0323, were selected for reference genome sequencing and assembly. *I. trifida* NCNSP0306 is a self-compatible inbred line derived from PI 540724 that was originally collected in Magdalena, Colombia; selfing of this accession has been sporadic and seed quantities are very limited. *I. triloba* NCNSP0323, is an inbred line derived from selfing of PI 618966 for at least five successive generations. PI 618966 was originally collected in Michoacan, Mexico. Seedlings of NCNSP0306 and NCNSP0323 were grown in the greenhouse under natural light supplemented with artificial light (16/8 h light/dark) and transferred to a dark room for 24 hours prior to sample collection to promote starch degradation. Young leaves were harvested and stored at -80°C prior to DNA extraction. To provide transcript evidence for genome annotation, completely opened flowers, young flower buds, young leaf, root and stem tissues were collected from both *I. trifida* and *I. triloba*, as well as callus tissues derived from flowers and stems of *I. trifida*. These tissues were flash frozen in liquid nitrogen and stored at -80°C until RNA extraction.

**DNA and RNA extraction**

Genomic DNA was extracted from young leaves using the QIAGEN DNeasy Plant Mini Kit following the manufacturer’s instructions (QIAGEN, Valencia, California). DNA quality was assessed via agarose gel electrophoresis and quantity was determined using a NanoDrop (Thermo Fisher Scientific, Waltham, MA). For all tissues except open flowers, total RNA was isolated from 100 mg of liquid nitrogen-ground plant tissue with the Qiagen RNeasy mini kit (Qiagen Inc., Valencia, CA); RNA was isolated from open flowers using a phenol extraction method^1^. RNA was treated with Turbo™ DNase (Ambion, Austin, TX) according to the manufacturer’s protocol and assessed for quantity and quality using a NanoDrop 1000 (Thermo Fisher Scientific, Waltham, MA) and Bioanalyzer 2100 with a RNA pico chip (Agilent Technologies, Santa Clara, CA).

## **Library construction, sequencing and data processing**

For NCNSP0306, paired-end genomic libraries with insert sizes of approximately 500 bp and 1 kb, and mate-pair libraries with inserts of 5, 10 and 15 kb were prepared using Illumina Genomic DNA Sample Preparation kit and Nextera Mate Pair Sample Preparation kit, respectively (Illumina, San Diego, CA). Mate-pair libraries (40 kb) were prepared for NCNSP0306 by Eurofins Genomics (Huntsville, AL). Similarly, paired-end (150 bp, 200 bp, 500 bp and 1 kb) and mate-pair (2, 5, 10, 15 and 40 kb) libraries were constructed for NCNSP0323. All genomic libraries were sequenced on the Illumina HiSeq 2500 platform in paired-end mode. PacBio libraries were prepared and sequenced on the PacBio RSII Sequencing System using the P5C3 chemistry (Pacific Biosciences, Menlo Park, CA). Strand-specific RNA-Seq libraries were constructed from total RNA using the protocol described in Zhong et al.^2^ and sequenced on the Illumina HiSeq 2500 system.

Illumina DNA sequence reads were first processed to collapse duplicated read pairs, defined as those having identical bases at positions of 14 to 80 in both left and right reads, into unique pairs. Reads were then processed with Trimmomatic^3^ (v0.32) to remove adaptor and low-quality sequences. For mate-pair sequences, the junction adaptor sequences were removed using the ShortRead package^4^ and then processed to correct errors with QuorUM^5^ (v1.1.0) with reads shorter than 40 nt discarded. PacBio reads were corrected with the Illumina reads using the MinHash Alignment Process (MHAP) pipeline^6^.

RNA-Seq data from eight *I. trifida* and six *I. triloba* tissues (**Supplementary Data 1**) were cleaned with Cutadapt^7^ (v1.8.3) using a quality trimming cutoff of 10 and a minimum read size of 31 nt. The cleaned RNA-Seq reads were aligned to the corresponding genome assemblies using TopHat2^8^ (v2.1.0) in strand-specific mode with a maximum intron size of 5 kb. Using Trinity^9^ (v2.1.1), RNA-Seq reads from each tissue were assembled separately using genome-guided transcript assembly with a minimum contig length of 500 bp and a maximum intron length of 5 kb.

**Supplementary Method 2. BioNano genome map generation**

High molecular weight DNA was prepared from healthy young leaves of *I. trifida* NCNSP0306 and *I. triloba* NCNSP0323 by Amplicon Express (Pullman, WA). The nicking endonuclease, Nt.BspQI, with an estimated frequency of 12 sites/100 kb was selected for DNA labeling. DNA was labeled using the IrysPrep® Reagent Kit (BioNano Genomics, San Diego, CA) according to the manufacturer's instructions, followed by ligation of the nicks with Taq ligase (New England BioLabs) in the presence of dNTPs, and staining with IrysPrep® DNA Stain (BioNano Genomics). A total of 56.1 and 74.7 Gb of data with molecule length exceeding 150 kb were generated using the IrysChip for *I. trifida* and *I. triloba*, respectively. These single molecules were used for *de novo* assemblies using IrysView^TM^ (BioNano Genomics, San Diego, CA), resulting in consensus maps with total length of 414.5 Mb and 450.7 Mb, and N50 length of 0.96 Mb and 1.37 Mb for *I. trifida*, and *I. triloba*, respectively. These consensus maps were then used to join the assembled scaffolds to form super-scaffolds using the “Sewing machine pipeline”^10^ with parameters of ‘--f_con 13 --f_algn 30 --s_con 8 --s_algn 90 -T 1e-8’.

**Supplementary Method 3. Genetic map construction and scaffold anchoring**

To construct a genetic map, we selected two genetically distinct accessions of diploid *I. trifida* (4653.22 and 4597.1) based on simple sequence repeat (SSR) markers and crossed these to generate a segregating F1 population from which two genetically distinct siblings (M9 and M19) selected using SSR markers were subsequently crossed to develop a segregating F1 mapping population. High-quality DNA was extracted from 212 progenies using the CTAB method^11^. A modified genotyping-by-sequencing (GBS) protocol optimized for highly heterozygous genomes was implemented for genotyping the M9 x M19 population. The DNA samples were initially quantified using an absorbance-based method (BioTek Synergy HTX Multi-Mode Plate Reader) and normalized to 50 ng/µl. A double-digest in an NEB CutSmart buffer was performed with *Cvi*AII at 25^°^C and then with *Tse*I at 65 ^°^C. The digested samples were purified with AMPure XP magnetic beads, quantified using a picogreen assay and then normalized to a concentration of 10 ng/μl before ligating to barcoded adapters, which were designed to include an 8-bp buffer sequence positioned upstream of the barcode sequence to ensure that the barcode sequences were within a high-quality base call region of the sequence read. Aliquots of the samples were pooled and then a secondary double-digest with *Cvi*AII and *Tse*I was performed to eliminate chimeric sequence ligations. The pools were again purified with AMPure XP magnetic beads, size-selected (250 to 450 bp fragments) on a Blue Pippin Prep system (Sage Science, USA), and amplified with 18 PCR cycles using the NEB Phusion high-fidelity polymerase. The resulting libraries were size selected again and then sequenced on an Illumina HiSeq 2500 platform. SNP calling from the GBS data using the *I. trifida* assembled scaffolds as the reference, genetic map construction and *I. trifida* scaffold anchoring were same as described in Zhou et al.^12^.

The resulting genetic map contained 15 linkage groups (LGs) with a total genetic length of 3,491.2 cM and 46,264 SNP markers, and an average 0.075 cM per SNP marker (**Supplementary Table 3** and **Supplementary Fig. 2**). The map was used to anchor and order the assembled scaffolds of *I. trifida*. Potential mis-scaffolding was detected using recombination frequencies between neighboring SNPs and interrogated further using the BioNano maps and mate-pair and paired-end reads. Thirteen initial *I. trifida* scaffolds were broken into 34 new scaffolds.

The *I. trifida* genetic map was also used to anchor the *I. triloba* scaffolds. The *I. trifida* SNP markers were converted into *I. triloba* positions based on unique best corresponding regions between the genomes of *I. trifida* and *I. triloba* using LAST^13^ (v869). Considering the nature of structural variations between the genomes of *I. triloba* and *I. trifida*, the *I. triloba* anchor points were ordered by not allowing changes of anchoring orders within a scaffold, breaking a scaffold by other scaffolds, or placing anchors from the same scaffold into different pseudomolecules.

**Supplementary Method 4. Transcriptome profiling**

**Biotic stresses of *I. trifida* and *I. triloba***

Detached leaf assays with two separate chemical elicitors, acibenzolar-S-methyl (BTH) and β-amino-n-butyric acid (BABA), were performed to simulate biotic stress responses in *I. trifida* and *I. triloba* (**Supplementary Data 1**). Prior to each treatment, fully expanded leaves of approximately uniform size were removed from greenhouse-grown plants of each species. To induce biotic stress, each chemical elicitor was dissolved in sterile water [BTH (100 μg/ml) and BABA (2 mg/ml)] and applied independently as a foliar spray treatment to three replicated leaves per species. A mock treatment with sterile water was performed in parallel. Treated leaves were placed individually onto moistened filter paper in sealed petri dishes and then transferred to a growth chamber with a 14 h photoperiod and constant 24°C temperature for incubation. Tissue samples from two leaves per species per treatment/control were collected at 24 hours post treatment, flash-frozen in liquid nitrogen and stored at -80°C.

**Abiotic stresses and hormone treatments of *I. trifida* and *I. triloba***

Abiotic stress responses and response to hormone treatments in both *I. trifida* and *I. triloba* were assessed with eight experimental treatments conducted with *in vitro*-grown plants (**Supplementary Data 1**). Plants from each species were grown in tubes containing a standard shooting medium (2.56 g/l modified MS salts with 1/2× ammonium nitrate and 1/2× potassium nitrate, 20 g/l sucrose, 6.5 g/l agar) and were maintained in the following growth chamber conditions: 14 h photoperiod and 28°C day/22°C night temperatures. After two clonal generations, three replicated plants from each species, each approximately uniform size, were selected for use in experimental treatments and controls. To induce cold- and heat-related stress responses, replicates from each species were transferred to a treatment chamber with a 14 h photoperiod and a 10°C day/4°C night or 35°C day/35°C night diurnal temperature cycle, respectively, for a 24 h period. Additional replicates from each species were maintained simultaneously under standard growth chamber conditions as experimental controls. For the remaining stress experiments, the standard shooting medium was modified as follows to include an additional ingredient related to each stress treatment: salt stress (150 mM NaCl), drought stress (260 mM Mannitol), abscisic acid stress (50 μM ABA), indole-3-acetic-acid stress (10 μM IAA), gibberellic acid stress (50 μM GA3), and 6-benzylaminopurine stress (10 μM BAP). Plants from each species were transplanted into a sterile polystyrene culture vessel (101.6 mm × 101.6 mm × 130 mm) containing a treatment medium. As a control, an additional set of replicates from each species were also transplanted into vessels containing the same standard shooting medium (i.e., salt, drought, and hormone controls). All experimental treatments and controls were incubated for 24 h. Following incubation, all above-ground organs (shoots, petioles, and leaves) from replicated plants were removed and pooled, flash-frozen in liquid nitrogen, and stored at -80°C prior to RNA isolation.

**Root development of cultivated sweetpotato**

Plants of sweetpotato cultivar ‘Beauregard’ were grown in a screenhouse at CIP in Lima, Peru between January and March 2016 in SOGEMIX® potting soil. Growing conditions included average temperature ranging from 23.9 ^o^C - 28.6 ^o^C with a mean temperature for the growing season of 26.7 ^o^C. Relative humidity (RH) ranged from 63.5% - 75.3% with a mean RH for the growing season of 68.9%. Photosynthetically active radiation (PAR) ranged from 47.7 μE - 187.4 μE with a mean PAR for the growing season of 122.5 μE. The pots were watered every two days to field capacity. Plants were fertilized using 22% N, 75% P and 25% K in two doses, provided as two grams per pot at one and two weeks after transplanting. The experiment was designed for destructive sampling of four biological replicates at 10, 20, 30, 40 and 50 days after transplanting (DAT) (**Supplementary Data 1**). At 10 and 20 DAT, total roots were sampled and classified as fibrous roots. At 30, 40 and 50 DAT, roots were sampled in two groups, fibrous and storage roots, based on diameter (>2.5 mm = storage roots; <2.5 mm = fibrous roots).

**RNA extraction, RNA-Seq library construction and sequencing**

Total RNA from all above samples except ‘Beauregard’ roots was isolated from 100 mg of liquid nitrogen-ground plant tissue with the Qiagen RNeasy mini kit (Qiagen Inc., Valencia, CA). Four isolations (i.e., from BTH and biotic control samples from both species) required additional cleaning and were processed further using the Qiagen RNeasy mini kit RNA cleanup protocol. For ‘Beauregard’ roots, RNA was extracted using Trizol following manufacturer’s instructions (Invitrogen, USA). All samples were treated with Turbo™ DNase (Ambion, Austin, TX) according to the manufacturer’s protocol and assessed for quantity and quality using a NanoDrop 1000 (Thermo Fisher Scientific, Wilmington, DE) and Bioanalyzer 2100 with RNA pico chip (Agilent Technologies, Santa Clara, CA). Strand-specific RNA-Seq libraries were constructed from total RNA using the protocol described in Zhong et al.^2^ and sequenced on an Illumina HiSeq 2500 platform.

**Differential gene expression analysis**

Raw RNA-Seq reads were processed by Trimmomatic^3^ (v0.32) to remove adapter, polyA/T tails and low quality (quality score < 20) sequences. Reads equal to or longer than 40 nt were kept and aligned to the SILVA rRNA database (release 111) (https://www.arb-silva.de/) to remove rRNA contamination. Cleaned reads of *I. trifida* and *I. batatas* cv. ‘Beauregard’ were aligned to the *I. trifida* genome and reads of *I. triloba* aligned to the *I. triloba* genome using HISAT2 (Ref. 14) allowing 4 mismatches. Raw counts for each gene were then derived and normalized to fragments per kilobase of exon model per million mapped reads (FPKM). DESeq^15^ was used to identify differentially expressed genes between different stress treatments and the corresponding mocks, and between different root types with a cutoff of adjusted *p*-value < 0.005, fold-change >2 and FPKM ≥ 3 in at least one sample. For heatmaps and the allele-specific expression analysis described below, two replicates for ‘Beauregard’ storage roots at 40 DAT were removed because Pearson’s correlation of FPKM values with at least one other replicate was less than 0.9.

***De novo* transcriptome assembly of hexaploid sweetpotato**

RNA-Seq data for *I. batatas* ‘Beauregard’ were *de novo* assembled to generate representative transcripts and peptides for comparative analyses. We utilized nineteen paired-end RNA-Seq libraries from ‘Beauregard’ (**Supplementary Data 1**), representing fibrous and storage root tissues sampled at different developmental stages and leaf tissues sampled from whole plants exposed to salt, heat, and mannitol. RNA-Seq reads were processed with Cutadapt^7^ (version 1.8.1) to remove adapter sequence, low-quality bases (quality scores <20), reads shorter than 31 nt, and poly(A) or poly(T) tails and *de novo* assembled using Trinity^9^ (version 2.2.0) with default parameters. Assembled transcripts longer than 500 bp were retained and for transcripts having multiple isoforms; only the longest was used for downstream analysis. Protein sequences of *I. batatas* were predicted from the assembled transcripts with TransDecoder (http://transdecoder.github.io).

**Supplementary Method 5. Quality assessment of the ‘haplotype-resolved’ hexaploid sweetpotato genome assembly**

Yang et al.^16^ recently reported an 836-Mb haplotype-resolved genome assembly of *I. batatas*, cv. Taizhong6, of which, 633 Mb were anchored to 15 pseudochromosomes based on sequence synteny to the *I. nil* genome^17^. To assess the quality of the ‘Taizhong6’ assembly, we first aligned the ‘Taizhong6’ assembly to the genomes of *I. nil*, *I. trifida* and *I. triloba* using LAST^13^ (v869), revealing a prevalence of “double-collinear” patterns, in which multiple sequences from the ‘Taizhong6’ assembly were syntenic to a single region in *I. nil* (**Supplementary Fig. 7**), *I. trifida* (**Supplementary Fig. 8**) or *I. triloba* (**Supplementary Fig. 9**), indicating the presence of redundancy in the ‘Taizhong6’ haplotype genome assembly. We then aligned bacterial artificial chromosome (BAC)-end sequences from sweetpotato cultivar Xu 781 (Ref. 18) to the ‘Taizhong6’ assembly. Surprisingly, alignments of BAC-end sequence pairs on a single chromosome of the ‘Taizhong6’ assembly revealed a median insert size of 3,036 kb (**Supplementary Fig. 10a**), significantly larger than the expected insert sizes of these BAC clones^18^ (101 kb). A parallel analysis using the *I. trifida* and *I. triloba* assemblies predicted an insert size distribution pattern consistent with the actual insert size (**Supplementary Fig. 10b** and **10c**). Furthermore, we aligned the full *Ib*T-DNA1 BAC sequence from cultivar Xu 781 (GenBank Acc#: KM113766) to the ‘Taizhong6’ assembly. Around 52.3% (41,639 out of 79,655 bp) aligned to chromosome 12 of the assembly scattered in a 1.2-Mb region (**Supplementary Fig. 11a**) and 31.2% aligned to other chromosomes or scaffolds. In contrast, good collinearity was found in a continuous region of the *I. trifida* and *I. triloba* genome assemblies (**Supplementary Fig. 11b** and **11c**). Mapping of the ‘Taizhong6’ mate-pair reads back to the assembled scaffold sequences within the ‘Taizhong6’ haplotype-resolved assembly identified regions with weak read support, many of which coincide with predicted breakpoints in scaffolds based on collinearity to the *I. trifida* assembly. For example, at least three regions that were not well supported by the mate-pair reads in the longest scaffold, scaffold3 (**Supplementary Fig. 12a**), overlapped with breakpoints suggested by the synteny of scaffold3 to *I. trifida* sequences on different chromosomes (**Supplementary Fig. 12b**).

The completeness of gene content in the ‘Taizhong6’ assembly was assessed using BUSCO^19^ (v3.0.2), which showed that only 73.4% of the core conserved plant genes were full-length in the assembly, with another 8.2% fragmented and 18.4% missing. Overall, these results indicate misassemblies and scaffolding errors in the *I. batatas* ‘Taizhong6’ assembly, which result in poor representation of sweetpotato genes and proteins. Downstream use of this genome assembly would be challenging for not only basic but also applied purposes.

**Supplementary Method 6. 10x Genomics data generation**

Two paired-end libraries were constructed from the high molecular weight genomic DNA of hexaploid sweetpotato cultivar ‘Tanzania’ and ‘Beauregard’ (one for each cultivar) using the 10x Genomics Chromium System following the manufacturer’s protocol (10x Genomics, Pleasanton, CA). The libraries were sequenced on an Illumina HiSeq X platform and the resulting linked reads were demultiplexed and mapped to the *I. trifida* and *I. triloba* genome assemblies using the 10x Genomics LongRanger pipeline. Read pairs that were marked as optical or PCR duplicates or not properly aligned were excluded from downstream analyses.

**Supplementary Method 7. Genome resequencing of hexaploid sweetpotatoes**

**Genome resequencing**

Sixteen accessions (**Supplementary Data 6**) were selected to constitute the MDP with the requirements of (i) having at least a unique trait of interest to farmers or consumers, and (ii) general adaptation to the important biotic and abiotic factors that negatively affect sweetpotato productivity in East and Central Africa^20^. Leaf tissue was ground to a fine powder using the FastPrep-24™ 5G tissue homogenizer (MP Biomedicals, Santa Ana, California) and DNA extracted from the leaf tissues following the protocols described in Dellaporta et al.^21^ and Mace et al.^22^ with modifications. Briefly, tissue was suspended in pre-warmed (65°C) CTAB buffer (200mM Tris-CL, 50mM EDTA, 2M NaCl, 2% CTAB and 3% β-mercapto-ethanol), mixed and heated at 65°C for 45 min prior to extraction with chloroform:isoamyl alcohol (24:1) and precipitation with sodium acetate and ethanol. Paired-end genomic libraries were prepared using Illumina’s Genomic DNA Sample Preparation kit and sequenced on the Illumina HiSeq 2500 system with paired-end mode and read length of 251 bp (Illumina, San Diego, CA).

**Putative aneuploidy identification**

Mean read depth per exon was calculated using the coverage function in BEDTools^23^ (v2.25.0) and BAM files after filtering for a minimum MAPQ score of 30, removal of PCR duplicates and indel realignment. We designed a framework for estimating the chromosome count of each sample using a binomial distribution. This test calculates the likelihood of the observed chromosome read depth as a function of the chromosome copy number for that sample, conditional on the total read count for that sample and the proportion of reads mapping to that chromosome in all other samples. We also used this framework to calculate the p-value of observing more than or equal to (in the case of a potential chromosome amplification), or less than or equal to (in the case of a potential chromosome deletion) the observed number of read mapping to that chromosome as a function of chromosome copy. Specifically, for each cultivar and each chromosome i, we define C_i_ and T_i_ be the number of reads mapping to chromosome i in this ‘foreground’ cultivar and all other cultivars combined (background cultivars), respectively. C = ∑C_i_ and T = ∑T_i_ were defined as the total read counts for the foreground cultivar and the remaining background cultivars combined, respectively. We modeled the probability distribution over reads observed in the cultivar sample in each window using the binomial distribution.

$prob\left( {x= C}_{i} \right| T_{i}, C, T,rcn) \sim Binomial \left( C_{i} \right| number of trials=C, p_{success}= rcn*{(T}_{i} /T) )$(1)

Where rcn is the relative copy number of the chromosome i in cultivar C versus the average of all the other cultivars. This allowed us to plot the probability of the observed number of reads as a function of rcn × 6 (to rescale to the assumption that the average of all other cultivars is hexaploid).

Where the observed number of reads C_i_ was less than the expected number of reads for rcn = 1, i.e. C*(T_i_ /T), we can calculate the probability of observing greater than or equal to the number of observed reads conditional on a range of rcn less than 1. We calculated rcn_max as the largest rcn for which this probability was significant at 1e-5 (i.e. p(x >= C_i_) < 1e-5). This had the property that the observed number of reads was significantly more than what would be expected for all rcn values less than rcn_max, but not for values of rcn greater than rcn_max. We investigated use of different p-value thresholds and found this had very little impact on the value of rcn_max, due to the very large number of reads observed, the transition from very significant to insignificant occurred extremely sharply as a function of rcn. We reported 6 × rcn_max to reflect the expectation that the average of all other cultivars (other than foreground cultivar) had a copy number of 6.

Similarly, where the observed number of reads C_i_ was greater than the expected number of reads for rcn = 1, i.e. C*(T_i_ /T), we can calculate the probability of observing fewer than or equal to the number of observed reads conditional on a range of rcn greater than 1. In this case we calculate rcn_min as the smallest rcn for which this probability was significant at 1e-5 (i.e. p(x <= C_i_) < 1e-5). We reported 6 × rcn_min.

To confirm the putative aneuploidy predicted based on read depth analysis, root tips were harvested from greenhouse-grown plants and chromosome spreads were prepared using protocols described in Braz et al.^24^. A minimum of five complete metaphase cells with well separated chromosomes were analyzed to determine the chromosome number of each accession. Chromosome images were captured using a QImaging Retiga EXi Fast 1394 CCD camera attached to an Olympus BX51 epifluorescence microscope and the final contrast of the images was processed using the Adobe Photoshop CS3 software.

**Supplementary Method 8. Carotenoid biosynthetic pathway analyses**

**Identification of SNPs associated with flesh color**

*Arabidopsis thaliana* proteins involved in the biosynthesis and cleavage of carotenoids (retrieved from PlantCyc [http://pmn.plantcyc.org]) were queried against the predicted proteomes of *I. trifida* and *I. triloba* using BLASTP. Only hits with a query coverage of at least 70% and an e-value of at most 1e-40 were considered for subsequent analysis (**Supplementary Data 7**). To identify SNPs/alleles significantly associated with orange flesh, bi-allelic variant sites in carotenoid-encoding gene models with no missing genotype data were identified using ANNOVAR^25^. The 16 MDP accessions were divided into white-fleshed and orange-fleshed groups, and at each variant site, the reference and non-reference alleles were counted. Fisher’s exact tests were performed to test for different proportions of reference and non-reference allele counts between white and orange-fleshed varieties.

**Allele-specific expression in genes associated with flesh color**

To test for allele-specific expression of SNPs in carotenoid biosynthetic pathway genes that had been identified as significantly associated with flesh color in the MDP accessions, SNP genotypes in the orange-fleshed cultivar ‘Beauregard’ were determined using 10x Genomics reads and allelic expression deviating from the genotype allele dosage was determined using RNA-Seq reads derived from a root development expression study with ‘Beauregard’ (see above). 10x Genomics reads from ‘Beauregard’ were aligned to the *I. trifida* genome as described for ‘Tanzania’. Alignments with MAPQ values less than 30 or flagged as PCR duplicates were removed. Subsequently, variants were called for ‘Beauregard’ using the same steps described for the MDP accessions, except ‘min-alternate-fraction’ for Freebayes^26^ was reduced from 0.1 to 0.05.

RNA-Seq read alignments were processed with the ‘SplitNCigarReads’ tool from GATK^27^ (v3.6.0), which splits alignments that span multiple exons into separate alignments in order to reduce inaccurate variant calls due to incorrectly spliced reads. Next, regions near indels were realigned as described for variant calls for the MDP accessions and allelic depths were calculated by running ‘mpileup’ from bcftools (v1.6; http://samtools.github.io/bcftools/bcftools.html), with parameters ‘-min-MQ 60, --redo-BAQ, --min-BQ 20’.

SNPs with missing/incomplete or homozygous genotypes for ‘Beauregard’ were removed. For each SNP, RNA-Seq samples with less than 30 total read depth were removed, and SNPs with only one replicate remaining after this filter were removed. Two-sided binomial tests were used to calculate significance for deviations from the allelic expression expected based on the genotype, and P-values from replicates of each experimental condition were combined using ‘sumlog’ from the metap package (<https://cran.r-project.org/web/packages/metap/index.html>) as described in Pham et al.^28^. Multiple testing correction was done using ‘p.adjust’ and the false discovery rate (FDR) method.

**Supplementary Method 9. Targeted sequence capture of species in the Batatas complex**

To perform targeted sequence capture, we first extracted a set of genes that were verified as being single copy in core eudicots including *Solanum tuberosum*, *Solanum lycopersicum*, *Arabidopsis thaliana*, *Thellungiella parvula*, *Carica papaya*, *Theobroma cacao*, *Populus trichocarpa*, *Fragaria vesca*, *Medicago trunculata*, *Glycine max*, *Vitis vinifera* as reported in *Amborella* Genome Project^29^. We then identified a subset of genes that were also single copy in *I. trifida* and *I. triloba* genome assemblies. RNA baits were designed to tile across exon sequences with 60 bp overlap between 120 bp biotinylated oligonucleotides (Arbor Biosciences, Ann Arbor, MI). In total, we targeted 1,953 exons distributed among 490 genes. Genomic DNA was isolated from fresh or dried leaf tissue of fifteen accessions representing the breadth of geographic and morphological diversity in the Batatas complex including three hexaploid (‘Jewel’, ‘Beauregard’ and ‘Tinian’) and two tetraploid (PI518474 and PI561261) *I. batatas* accessions, using a modified CTAB protocol^30,31^. Libraries were prepared using the KAPA HTP DNA Library Kit for Illumina (Kapa Biosystems, Wilmington, MA). Four barcoded DNA libraries were pooled in equal concentration, and RNA baits were hybridized to pooled DNA libraries following guidelines in the MyBaits protocol (version 3). The biotinylated baits bound to DNA were captured with streptavidin beads (ThermoFisher Scientific, Waltham, MA). The resulting enriched libraries were sequenced on the Illumina NextSeq platform with 150 bp paired-end reads.

Raw reads were processed using Trimmomatic^3^ (v0.36) to trim adapter and barcode sequences as well as low quality ends. Reads shorter than 40 nt after trimming were discarded. Trimmed reads were assembled *de novo* using Trinity^9^ (v2.0.6). The assembled contigs were removed from the analysis if more than one contig matched the same exon in the reference exon set from *Ipomoea trifida* ITR from Hirakawa et al.^32^. Species tree estimations are typically based on a set of single-haplotype ortholog alignments. Our exon baits were designed for 490 genes that were single copy in eudicot genomes including *I.* *trifida*^32^, but based on read mapping in ‘Beauregard’ and the 16 hexaploid MDP accessions to the *I.* *trifida* genome (**Supplementary Fig. 16a**), most of these single-copy genes have retained three copies in hexaploid *I. batatas* genomes. As seen in the high frequency of simplex SNP loci (**Supplementary Fig. 14**), however, haplotype diversity among homeologous gene copies is generally low across hexaploid *I. batatas* genomes. For the phylogenetic analyses we only included *I. batatas* genes for which we assembled a single haplotype (i.e. nullplex genotypes) or two highly similar haplotypes (simplex) that were found to form a clade in gene trees. After removing genes exhibiting more haplotype diversity, 85 genes were found to be single-haplotype in the *I. batatas* samples. Exon sequences for theses 85 genes were also extracted from the diploid *I. trifida* (ITRk^32^ and NCNSP0306) and *I. triloba* (NCNSP0323) genome assemblies, and the mapped reads from the 16 MDP, ‘Tanzania’ and ‘Taizhong6’^16^ *I. batatas* accessions. Multiple haplotypes were assembled for multiple genes extracted from each of the *I. batatas* accession read sets. After filtering as described above, only six of the MDP accessions (‘Ejumula’, ‘NASPOT 5/58’, ‘NASPOT 7’, ‘Magabali’, ‘Wagabolige’ and ‘Mugande’) and ‘Taizhong6’^16^ retained at least 30 nullplex or single-clade haplotype genes. All other accessions were excluded from further analyses.

**Supplementary Fig. 1 K-mer distribution of Illumina genome sequencing reads of *I. batatas* ‘Taizhong6’, *I. trifida* NCNSP0306, and *I. triloba* NCNSP0323.** K-mer distribution of *I. batatas* ‘Taizhong6’ was generated using Illumina genome sequencing reads reported in Yang et al. (2017).

**
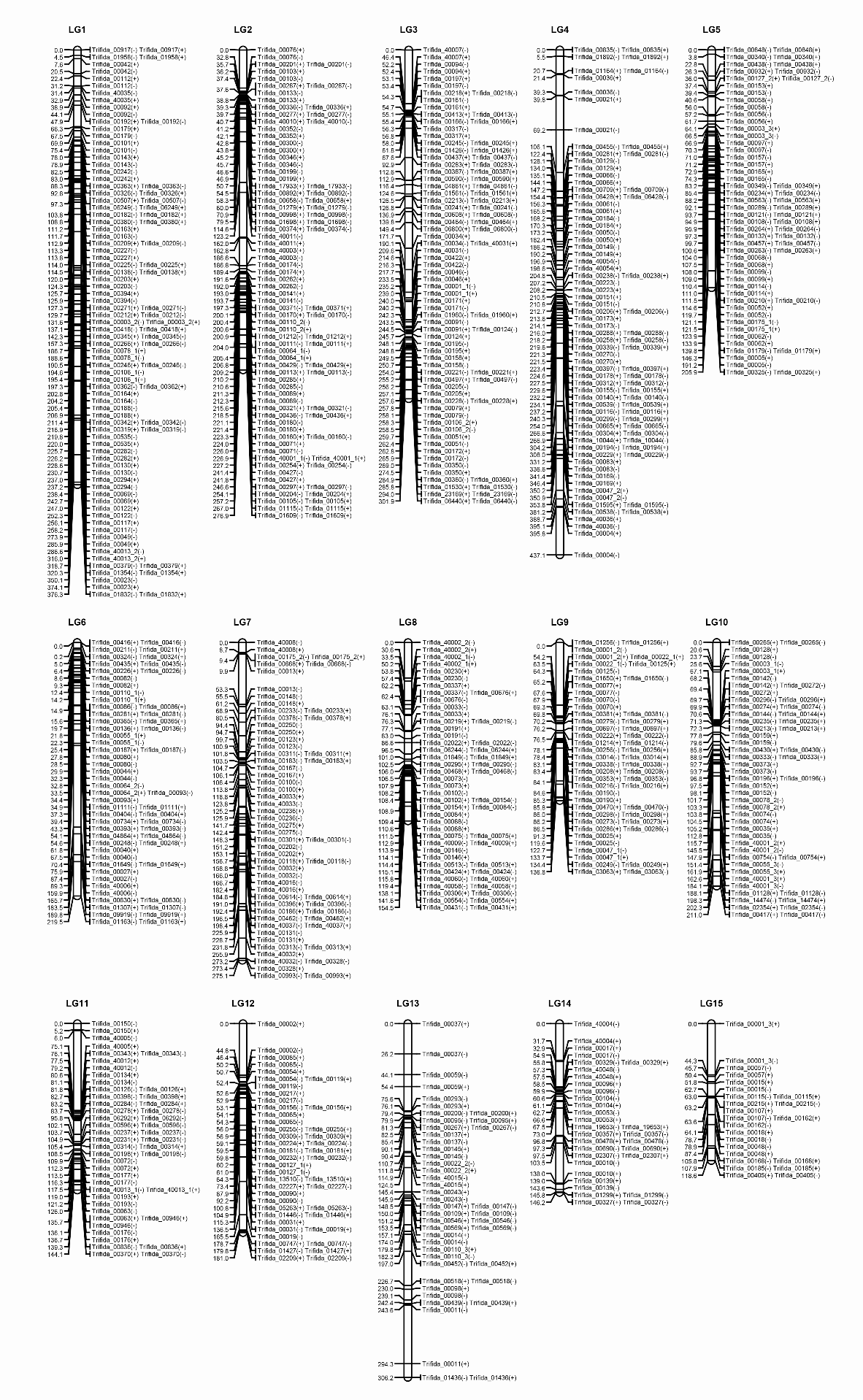
**

**Supplementary Fig. 2 Genetic map of *I. trifida*.** Positions in centimorgan are labeled on the left. The first and last markers from a scaffold are indicated by “+” and “-”, respectively.

**Supplementary Fig. 3 Phylogeny of Kunitz-type trypsin inhibitor (KTI) proteins in *I. trifida*, *I. triloba* and other representative species.** The phylogenetic tree was built using the ETE3 workflow (Huerta-Cepas et al., 2016). Multiple sequence alignment of 285 full-length KTI protein sequences was performed using MUSCLE (https://www.ebi.ac.uk/Tools/msa/muscle/), followed by gap removal with trimAl (http://trimal.cgenomics.org/). Phylogenetic tree of KTIs was constructed using RAxML (https://sco.h-its.org/exelixis/web/software/raxml/) with default parameters and 100 bootstraps. The tree was rooted using KTIs from Amborella. Bootstrap values are shown next to the branches. Branches corresponding to partitions reproduced in less than 50% bootstrap replicates are collapsed. KTIs from *I. trifida*, *I. triloba*, *I. nil*, potato, tomato, grapevine, Arabidopsis, rice and Amborella are labeled in orange, brown, cyan, green, blue, purple, light green, pink and black, respectively. The two sporamin genes that were highly expressed in sweetpotato storage roots are indicated by red dots. The subgroup that is specific to *I. trifida* and *I. triloba* is highlighted in orange.


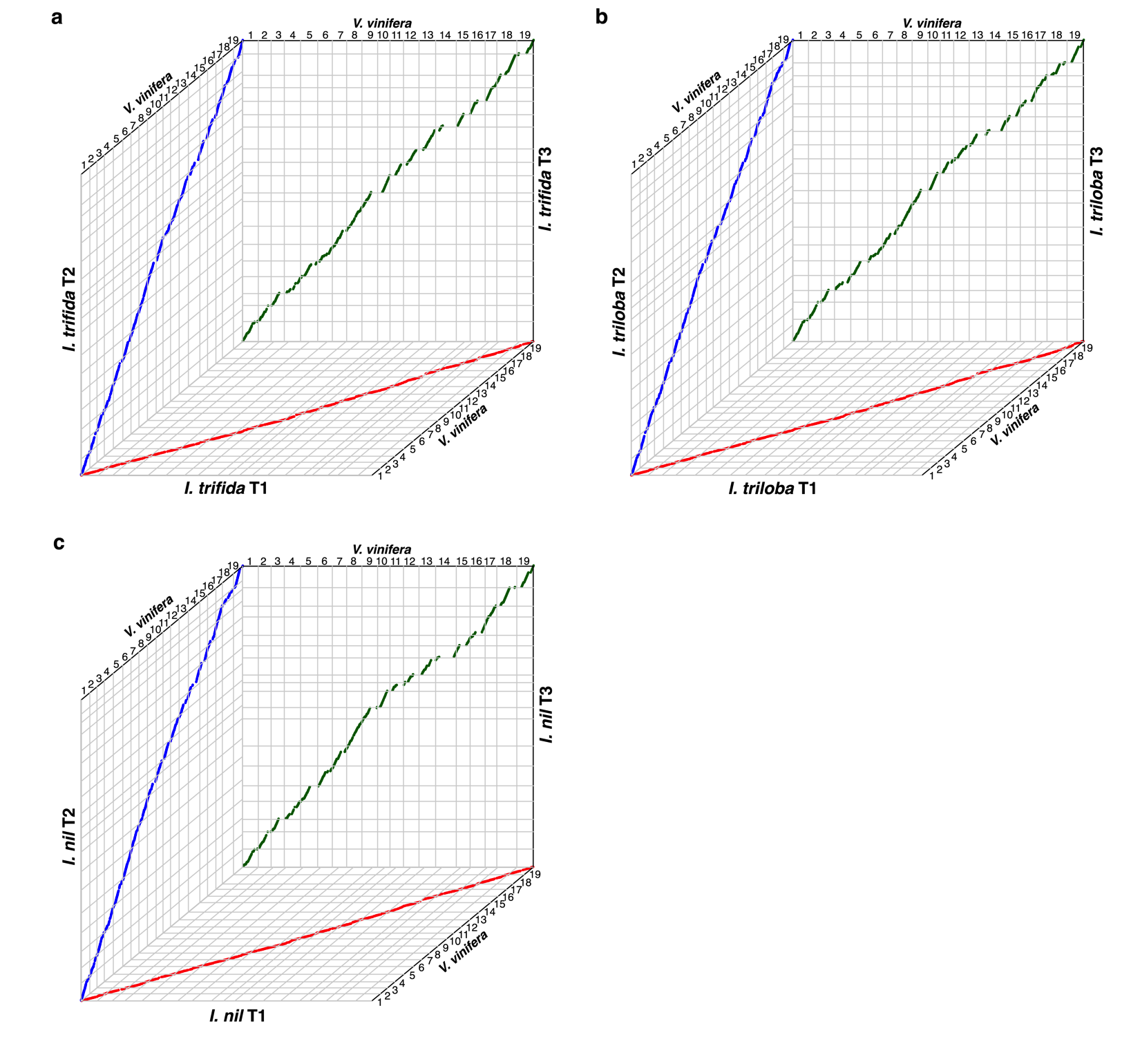


**Supplementary Fig. 4 Three partitioned non-overlapping ‘subgenomes’, each represented by one axis in the three-dimensional plot, of *I. trifida* (a), *I. triloba* (b) and *I. nil* (c).** The partition to ‘subgenomes’ was based on the alignments of multiple genome regions of *I. trifida,* *I. triloba* or *I. nil* to single grape genome segments.

**Supplementary Fig. 5 Syntenic dotplots between the genomes of *I. triloba* and *I. trifida* (a), and between *I. nil* and *I. trifida* (b).**

Continue on next page.

**Supplementary Fig. 5**

**
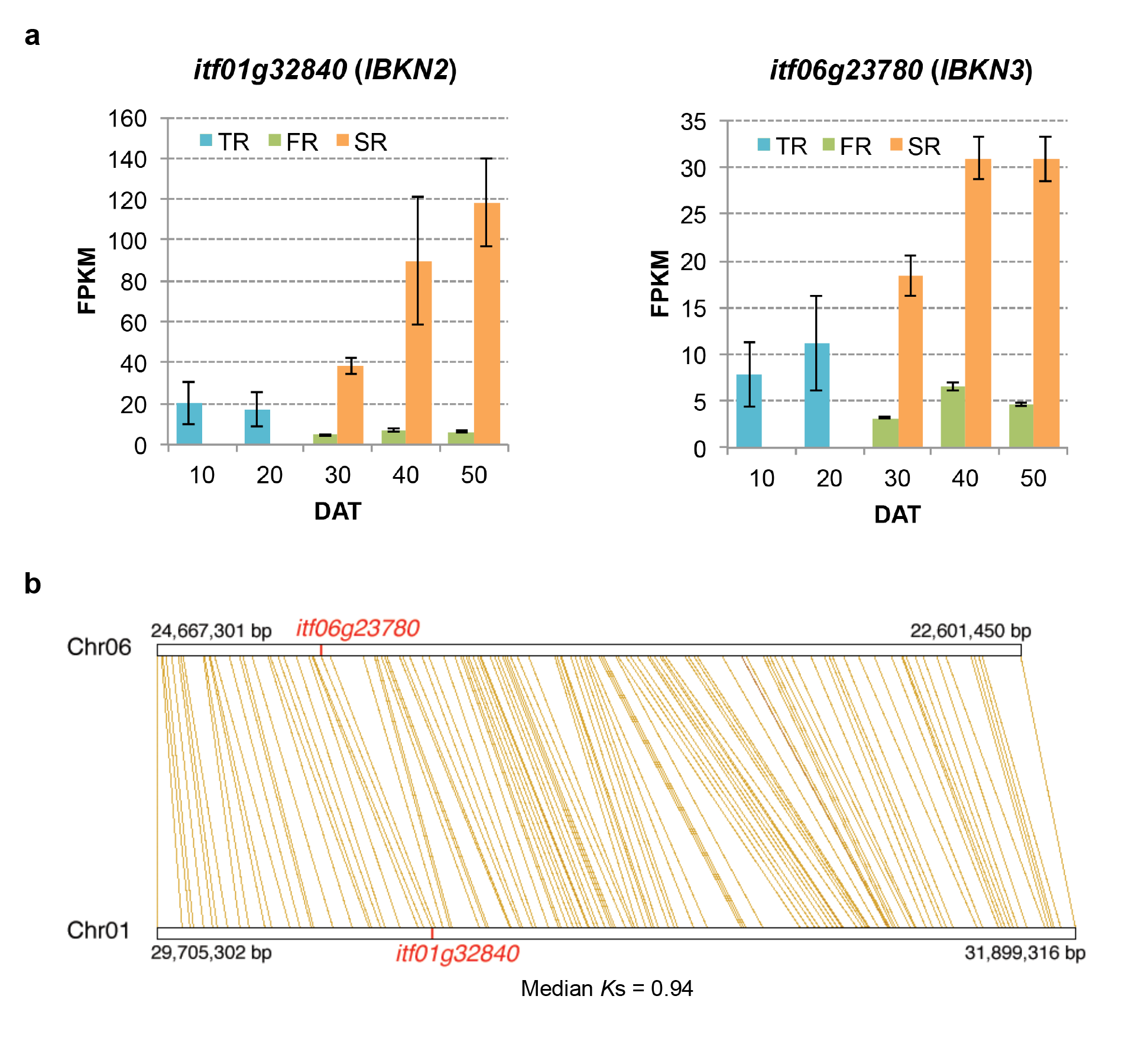
**

**Supplementary Fig. 6 Duplicated sweetpotato Knotted-like homeobox genes due to the *Ipomoea* WGT event. a**, Expression of *IBKN2* and *IBKN3* in ‘Beauregard’ root tissues. DAT, days after transplanting; TR, total undifferentiated roots; FR, fibrous roots; SR, storage roots. FPKM, fragment per kilobase of exon model per million mapped reads. Error bars represent standard errors of three or four biological replicates. **b**, A ~2 Mb syntenic homologous block between *I. trifida* chromosomes 1 and 6. Pairs of syntenic paralogous genes are connected by lines. The *I. trifida* orthologs of *IBKN2* (*itf01g32840*) and *IBKN3* (*itf06g23780*) are indicated by red bars.

**Supplementary Fig. 7 Syntenic dotplots of the *I. batatas* Taizhong6 haplotype assembly (*x*-axis) and the *I. nil* genome (*y*-axis).** The zoomed-in insert shows an example of local double collinear pattern.

**Supplementary Fig. 8 Syntenic dotplots of the *I. batatas* Taizhong6 haplotype assembly (*x*-axis) and the *I. trifida* NCNSP0306 assembly (*y*-axis).** The zoomed-in insert shows an example of local double collinear pattern.

**Supplementary Fig. 9 Syntenic dotplots of the *I. batatas* Taizhong6 haplotype assembly (*x*-axis) and the *I. triloba* NCNSP0323 assembly (*y*-axis).** The zoomed-in insert shows an example of local double collinear pattern.


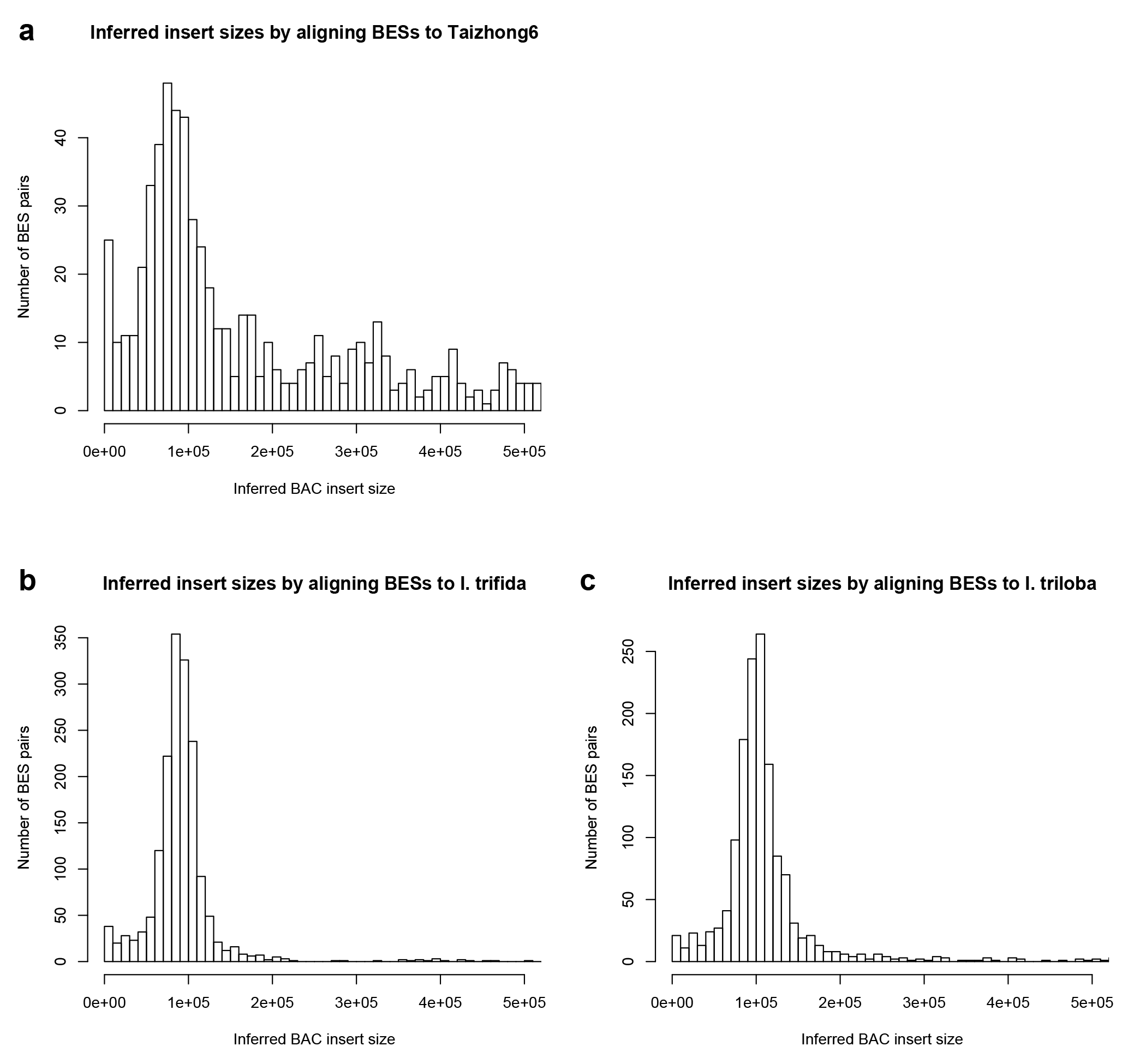


**Supplementary Fig. 10 Alignment of ‘Xu 781’ BAC-end sequences (BESs) to the *I. batatas* Taizhong6 assembly. a-c,** Distribution of insert sizes of the BAC clones inferred from the paired BESs mapped to the assemblies of *I. batatas* Taizhong6 (**a**), *I. trifida* NCNSP0306 (**b**) and *I. triloba* NCNSP0323 (**c**).

**Supplementary Fig. 11 Alignment of the *Ib*T-DNA1 BAC sequence to the Taizhong6 assembly. a,** Syntenic dotplots of the *Ib*T-DNA1 BAC (GenBank Acc#: KM113766) and the chromosome 12 and scaffold 14997 of *I. batatas* Taizhong6. **b**-**c,** Syntenic dotplots between the *Ib*T-DNA1 BAC and the chromosome 4 of *I. trifida* (**b**) and *I. triloba* (**c**).


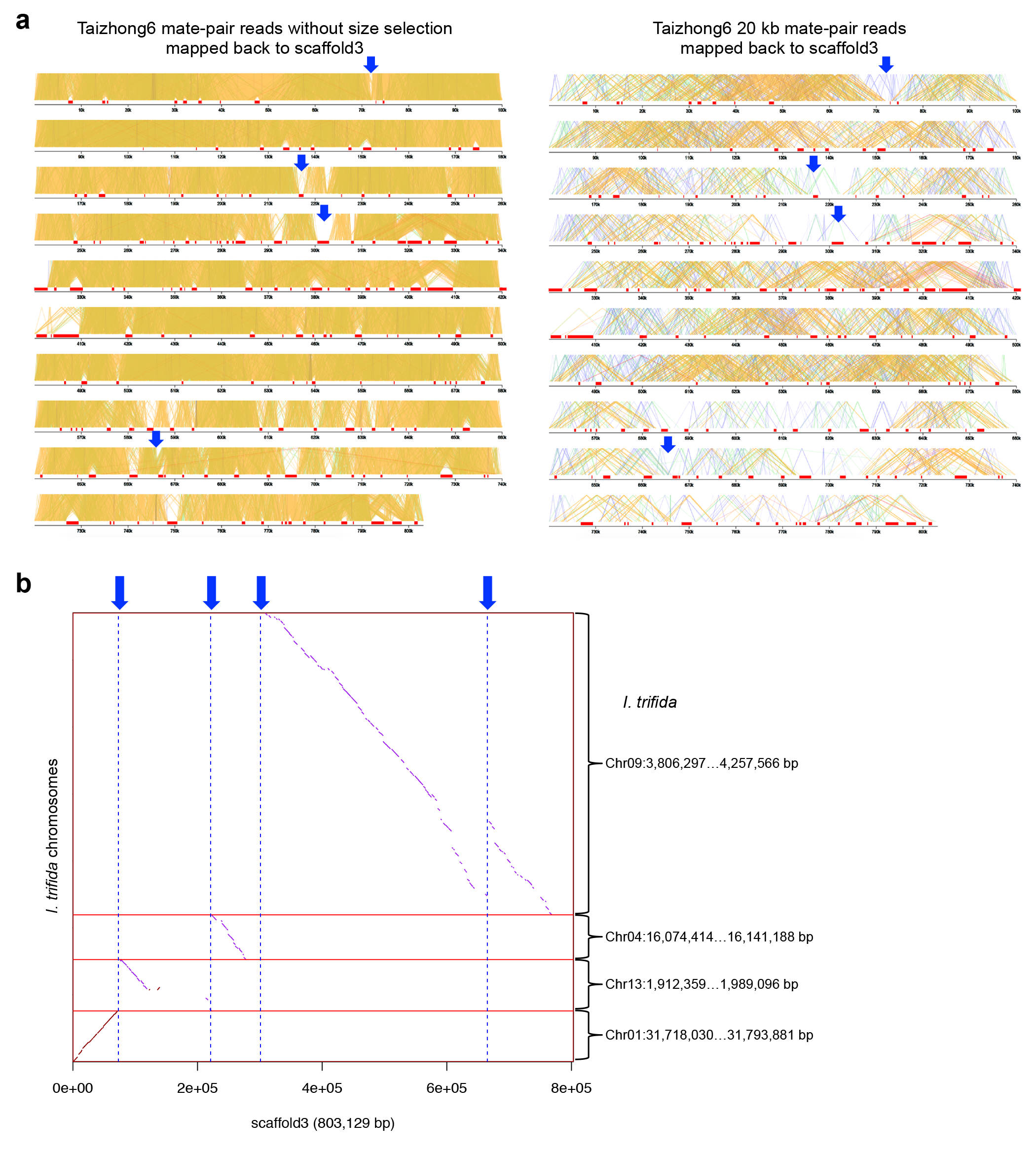


**Supplementary Fig. 12 Mapping of mate-pair reads back to the *I. batatas* ‘Taizhong6’ scaffold3. a,** Alignment of ‘Taizhong6’ mate-pair reads from libraries without size selection (left) and 20-kb insert libraries (right). Blue arrows point to assembled regions with weak or no read support. Uniquely aligned read pairs with correct insert size and insert size larger or smaller than expected are indicated by orange and red or blue lines, respectively. Reads aligned to multiple locations are shown by green lines. Red bars indicated the gaps in the scaffolds. **b,** Syntenic dotplots of *I. batatas* ‘Taizhong6’ scaffold3 compare to the *I. trifida* NCNSP0306 chromosomes. Blue arrows point to regions in the *I. batatas* assembly weakly supported by the mate-pair reads as show in (**a**).


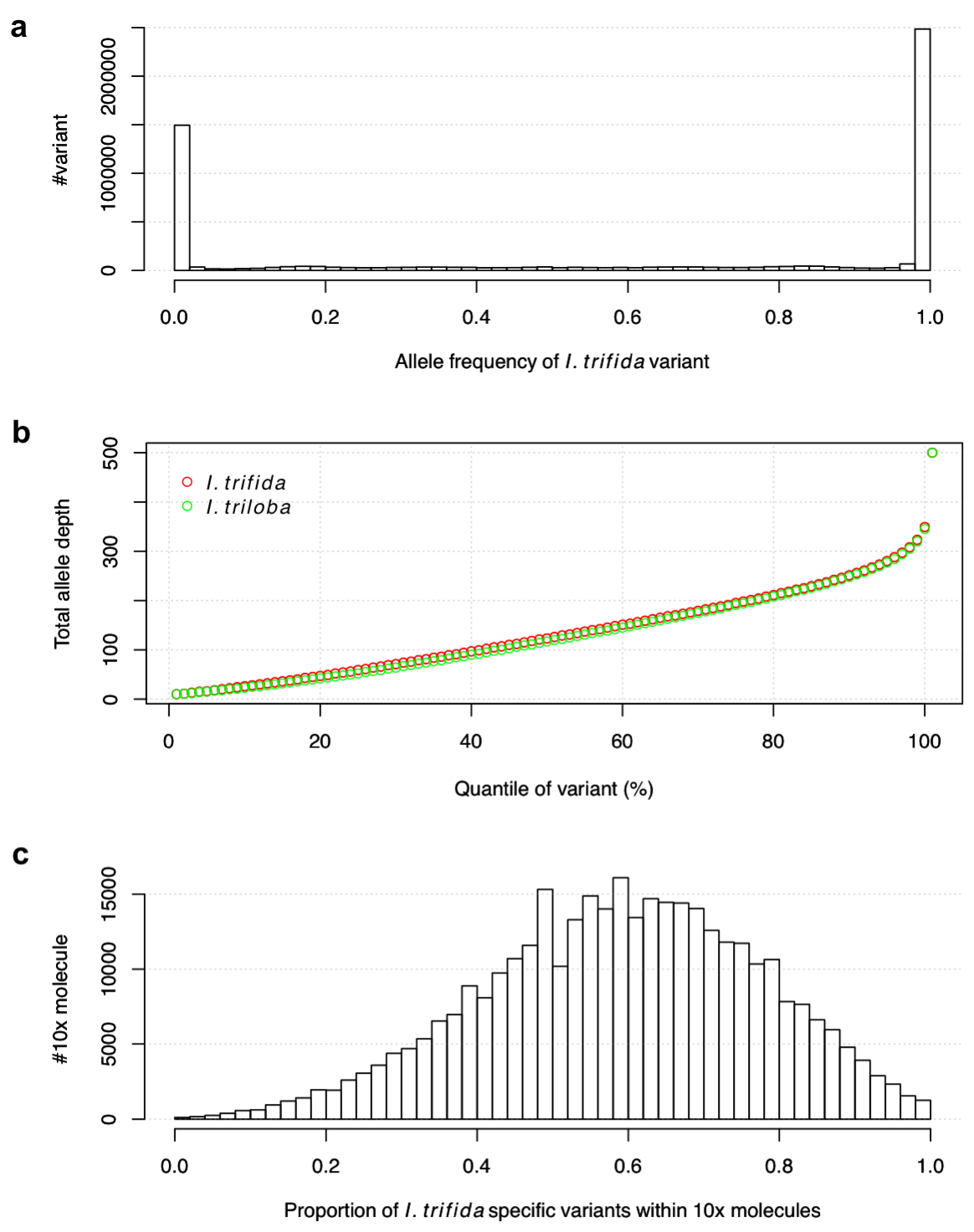


**Supplementary Fig. 13 Statistics of the allele frequencies of the *I. trifida*- and *I. triloba-*specific polymorphic sites in ‘Tanzania’**. **a**, Distribution of allele frequencies of the *I. trifida-*specific variants. The allele frequencies were calculated as the allele depth divided by the total depth using the 10x Genomics sequencing data. The variants of allele frequency of 0.0 and 1.0 are homozygous sites comprising *I. triloba-*specific and *I. trifida*-specific alleles, respectively. **b**, Distribution of the total allele depth of the homozygous sites comprising *I. trifida* specific and *I. triloba* specific alleles, respectively. The two distributions are similar indicating no copy number difference between the *I. trifida* specific and *I. triloba* specific homozygous sites. **c**, Distribution of the proportion of *I. trifida* specific variants within 10x molecules. The variant proportions were calculated as the number of sites representing the *I. trifida* specific variants divided by the total number of variant sites (≥10) within the 10x molecule.


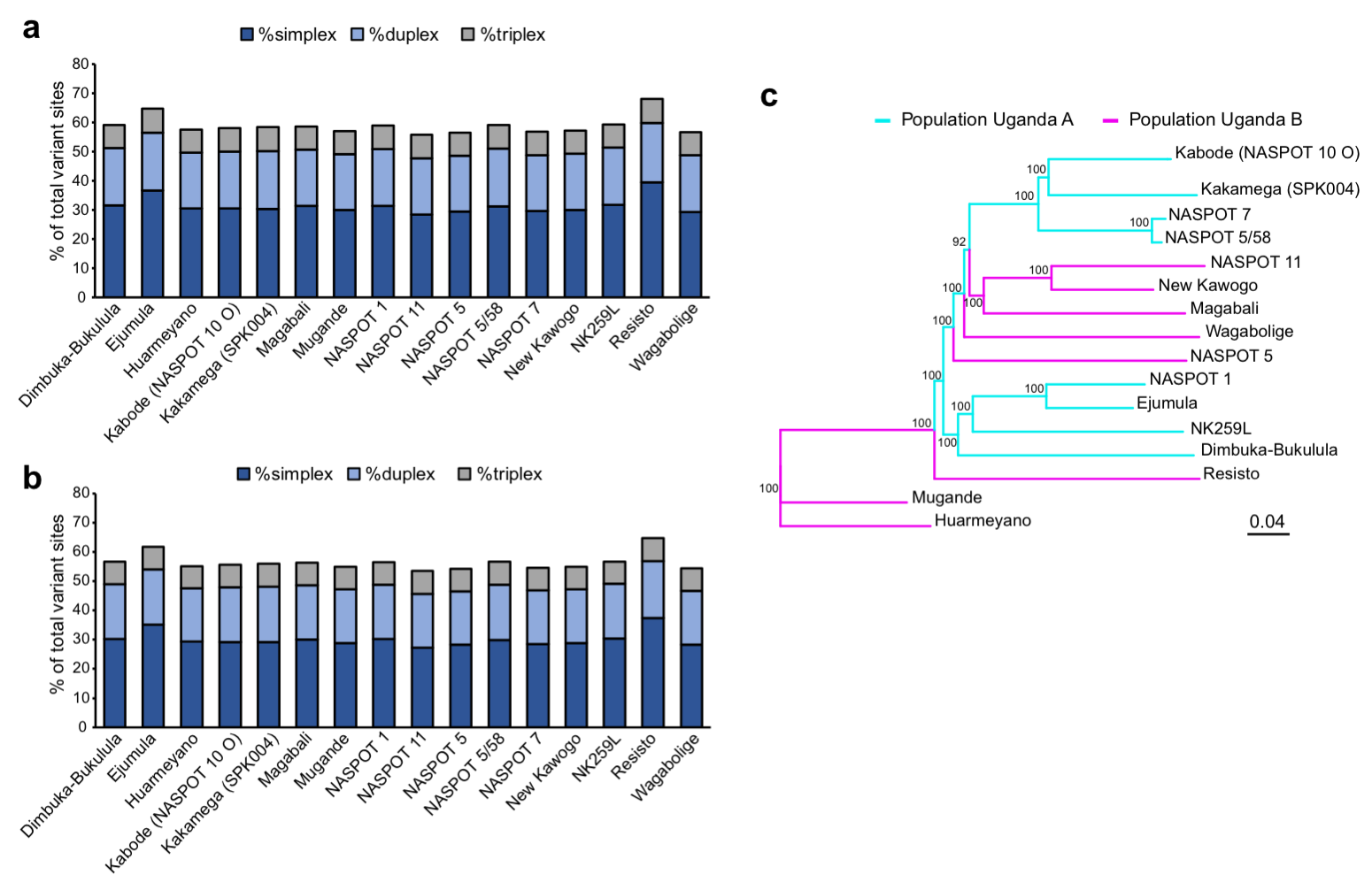


**Supplementary Fig. 14 Genetic diversity of the 16 MDP accessions. a**-**b,** Proportion of dosage classes for variants called for the MDP accessions based on alignments of whole-genome sequence reads against the *I. trifida* (**a**) and *I. triloba* (**b**) genome assemblies. Both A/B/B/B/B/B or A/A/A/A/A/B genotypes were considered simplex, and both A/A/B/B/B/B or A/A/A/A/B/B genotypes were considered duplex. **c,** Neighbor-joining tree of the MDP accessions based on SNPs detected using the *I. triloba* genome as the reference. Population membership based on a previous SSR analysis is indicated by branch colors. Numbers at nodes indicate the percentage of 1,000 bootstrap replications that support each clade. Huarmeyano was used as an outgroup for rooting.

**Supplementary Fig. 15 Population structure of the 16 MDP accessions. a,** PCA analysis of the MDP accessions. **b,** Population structure of the MDP accessions. Each color represents one population, each accession is represented by a vertical bar, and the length of each colored segment in each vertical bar represents the proportion contributed by ancestral populations.


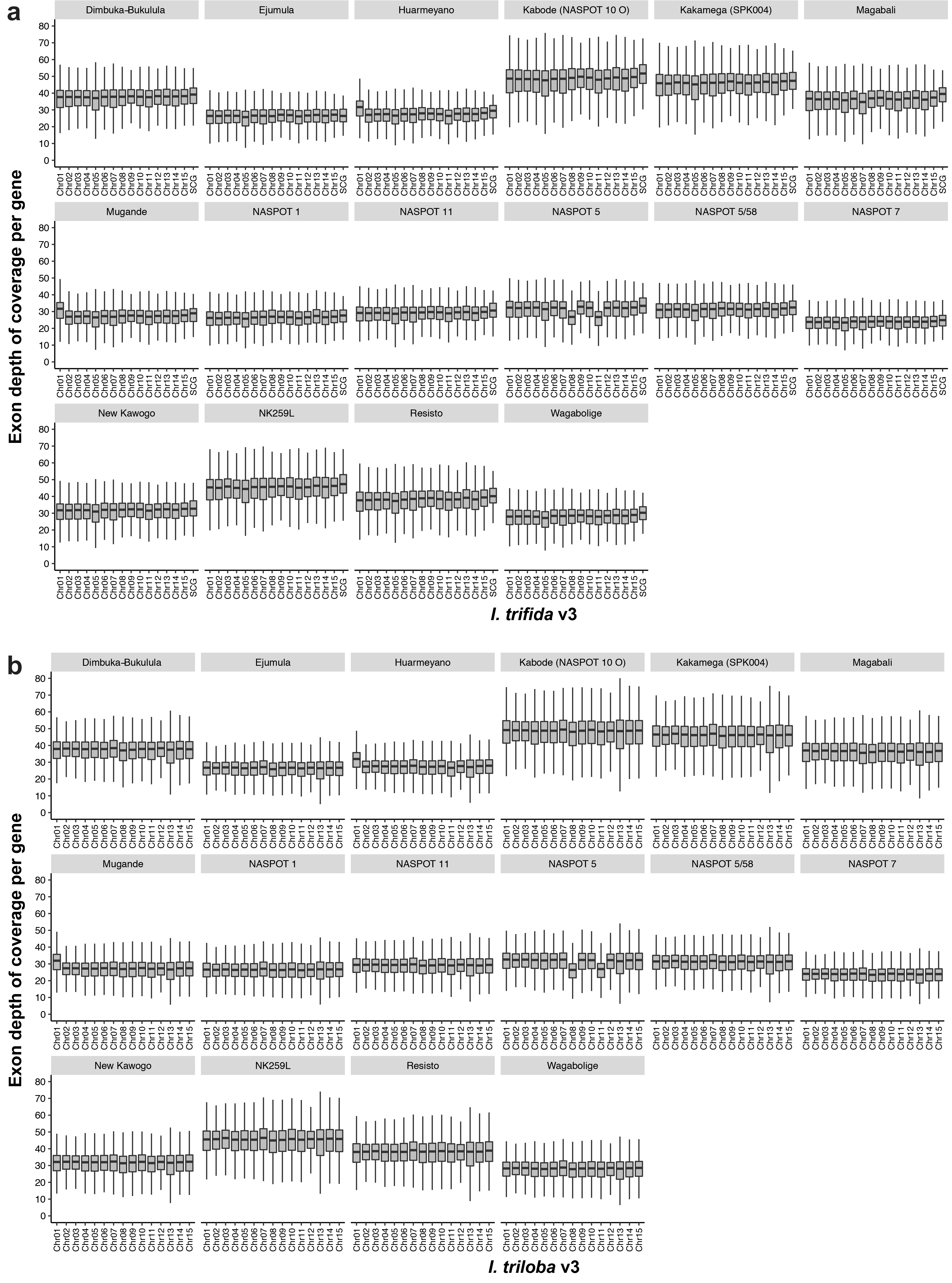


**Supplementary Fig. 16 Distribution of mean read depth per exon for each chromosome among 16 MDP accessions based on alignments to *I. trifida* (a) and *I. triloba* (b) reference genomes.** SCG: single-copy genes used in the target sequence capture analysis. For each box plot, the lower and upper bounds of the box indicate the first and third quartiles, respectively, and the center line indicates the median.

**Supplementary Fig. 17 Log likelihood of observed read-depth per chromosome calculated using a binomial model.**For each sample in turn the binomial probability of a read mapping to the given chromosome using all the remaining samples was calculated. This binomial probability is multiplied by the relative chromosome copy number (i.e. chromosome count divided by 6).

**Supplementary Fig. 18** Chromosome counting of Mugande and Tanzania revealing 90 chromosomes in each accession. Bars represent 10 µm.

**Supplementary Fig. 19 Allele specific expression in ‘Beauregard’ root tissues of SNPs significantly** **enriched in the orange-fleshed MDP accessions.** Allelic depth as proportions of total depth for whole-genome sequencing (left) and RNA-Seq (right) of different ‘Beauregard’ root tissue. Genotypes called by Freebayes are indicated above each chart. Allelic depth proportions based on means of two or more replicates are shown with significance for allele specific expression indicated. SNPs not shown here include those that are intronic or absent from the ‘Beauregard’ SNP dataset. SNP at the Chr03:3,120,243 was in phase with SNP at the Chr03:3,120,259 in 100% of >100 overlapping ‘Beauregard’ genomic reads and was manually annotated as 0/0/0/1/1/1. n.s., not significant; *, P<0.05; **, P<0.001.

**
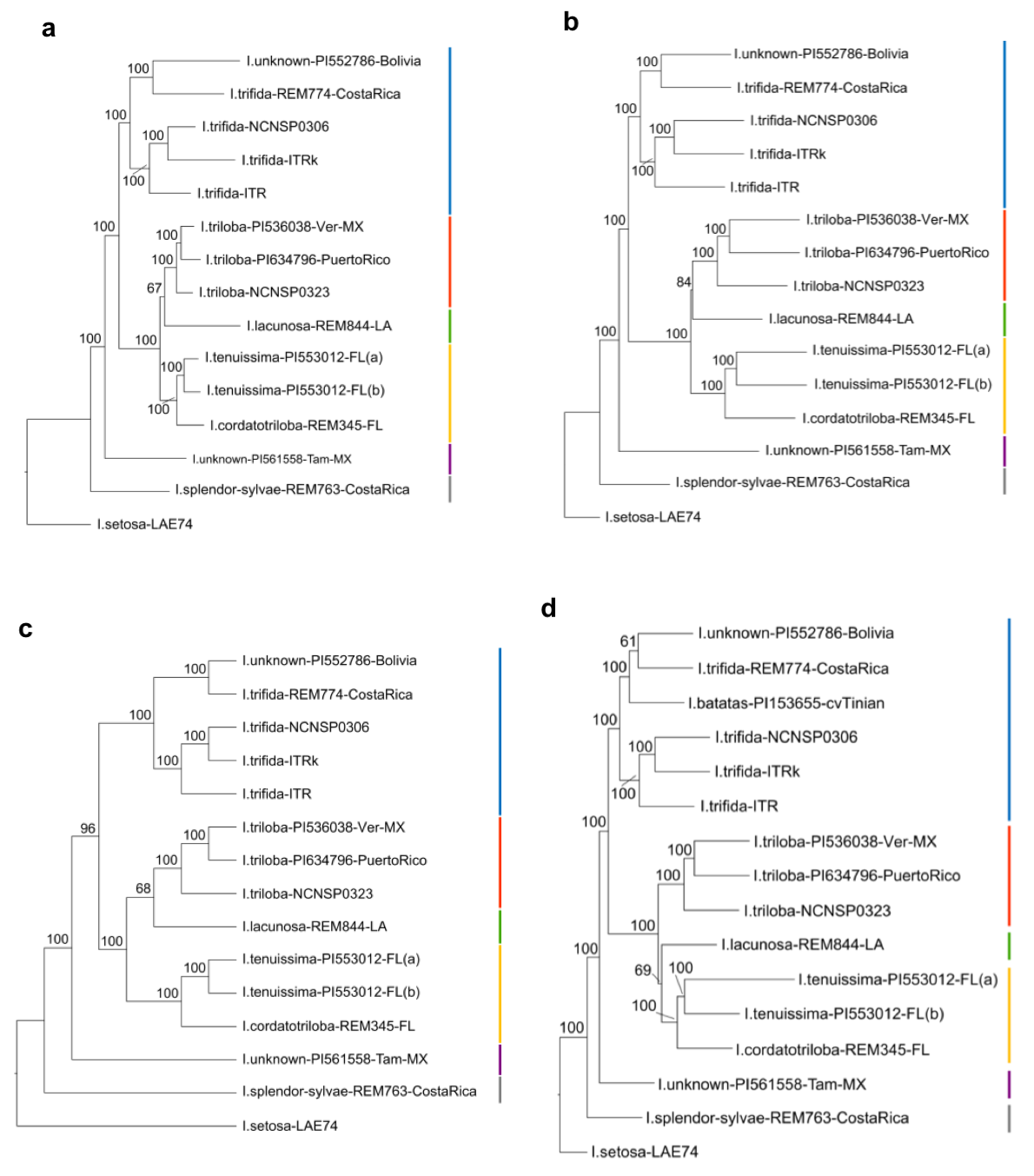
**

**Supplementary Fig. 20 Phylogenetic relationships among the diploid wild relatives of sweetpotato.** Shown are phylogenetic trees constructed using 361 genes with the concatenated dataset using RaxML (**a**), ASTRAL-II (**b**), and SVDQuartets (**c**). A PhyloNet tree including *I. batatas* (‘Tinian’) but estimated without reticulations is identical to the ASTRAL-II tree estimated on the same alignment and places the Tinian within the *I. trifida* clade (**d**). Color bars at the right of each tree denote the six inferred major lineages.

**Supplementary Table 1. Summary of the genomic sequencing reads**

| **Library** | | | **Average insert length (bp)** | **Raw** | | **Cleaned** | | |
| --- | --- | --- | --- | --- | --- | --- | --- | --- |
| **Type** | | **Name** |  | **Average read length (bp)** | **No. of bases** | **Average read length (bp)** | **No. of bases** | **Depth** |
| ***I. trifida*** | |  |  |  |  |  |  |  |
| **Illumina** | **Paired-end** | 500bp_1 | 454 | 160 | 19,755,530,240 | 156 | 18,294,336,035 | 35 |
|  |  | 500bp_2 | 515 | 160 | 21,662,094,080 | 154 | 19,105,508,952 | 36 |
|  |  | 1kb | 720 | 160 | 35,287,946,240 | 154 | 30,861,049,402 | 59 |
|  | **Mate-pair** | 5kb | 3,384 | 160 | 23,756,865,280 | 120 | 10,267,845,053 | 20 |
|  |  | 10kb | 7,913 | 160 | 31,187,171,840 | 116 | 10,926,840,626 | 21 |
|  |  | 15kb | 15,500 | 160 | 47,645,696,640 | 114 | 9,696,441,703 | 18 |
|  |  | 40kb | 32,772 | 30-100 | 22,433,505,116 | 90 | 5,603,954,875 | 11 |
|  | **Total** | - | - | - | 201,728,809,436 | - | 104,755,976,646 | 199 |
| **PacBio** |  | - | - | 4,468 | 11,909,256,312 | 3,225 | 5,867,994,415 | 11 |
| ***I. triloba*** | |  |  |  |  |  |  |  |
| **Illumina** | **Paired-end** | 150bp | 162 | 101 | 48,924,989,032 | 98 | 28,299,389,500 | 57 |
|  |  | 200bp | 204 | 101 | 40,317,947,000 | 98 | 13,247,071,880 | 27 |
|  |  | 500bp_1 | 207 | 101 | 4,882,991,248 | 99 | 3,363,784,139 | 7 |
|  |  | 500bp_2 | 510 | 101 | 33,174,385,866 | 100 | 28,778,638,321 | 58 |
|  |  | 1kb | 714 | 150 | 35,708,590,800 | 146 | 32,479,802,107 | 65 |
|  | **Mate-pair** | 2kb | 1,881 | 101 | 22,332,288,366 | 86 | 9,522,646,147 | 19 |
|  |  | 5kb | 4,374 | 101 | 21,778,758,068 | 86 | 8,867,701,122 | 18 |
|  |  | 10kb | 7,888 | 150 | 16,452,249,000 | 116 | 8,500,274,191 | 17 |
|  |  | 15kb | 12,762 | 150 | 20,168,044,500 | 111 | 8,783,475,429 | 18 |
|  |  | 40kb | 44,178 | 30-100 | 10,655,743,809 | 88 | 2,263,272,792 | 5 |
|  | **Total** | - | - | - | 254,395,987,689 | - | 144,106,055,628 | 291 |
| **PacBio** |  | - | - | 2,445 | 7,124,598,193 | 1,526 | 2,293,821,948 | 5 |

Sequencing depth was calculated based on the estimated genome sizes of *I. trifida* (526,485,521 bp) and *I. triloba* (495,937,308 bp).

**Supplementary Table 2. Summary statistics of I. trifida and I. triloba genome assemblies**

|  | | **Scaffold*** | | **Contig** | |  |  |
| --- | --- | --- | --- | --- | --- | --- | --- |
|  |  | **Size (bp)** | **Number** | **Size (bp)** | **Number** |  |  |
| ***I. trifida*** | Longest | 8,902,984 | 1 | 1,067,799 | 1 |  |  |
|  | N50 | 1,237,020 | 74 | 65,820 | 1,517 |  |  |
|  | N90 | 22,554 | 1,020 | 4,204 | 10,951 |  |  |
|  | Total | 461,997,559 | 30,394 | 433,249,235 | 44,843 |  |  |
| ***I. triloba*** | Longest | 19,833,707 | 1 | 313,171 | 1 |  |  |
|  | N50 | 6,861,300 | 24 | 36,931 | 3,562 |  |  |
|  | N90 | 2,051,804 | 73 | 8,536 | 12,673 |  |  |
|  | Total | 457,835,428 | 4,008 | 437,549,984 | 31,272 |  |  |

*Scaffolds shorter than 500bp were excluded from the assemblies.

**Supplementary Table 3. Summary of anchored *I. trifida* and *I. triloba* scaffolds based on the genetic map**

| **LG** | **No. of scaffolds** | | **Anchored** | | **Oriented** | |
| --- | --- | --- | --- | --- | --- | --- |
|  | **Anchored** | **Oriented** | **Length (bp)** | **%** | **Length (bp)** | **%** |
| ***I. trifida*** |  |  |  |  |  |  |
| **LG1** | 51 | 26 | 32,197,286 | 6.97 | 26,431,369 | 5.72 |
| **LG2** | 47 | 19 | 27,567,708 | 5.97 | 19,936,579 | 4.32 |
| **LG3** | 45 | 21 | 28,575,729 | 6.19 | 25,001,888 | 5.41 |
| **LG4** | 45 | 18 | 32,468,527 | 7.03 | 25,873,346 | 5.60 |
| **LG5** | 32 | 14 | 26,134,527 | 5.66 | 20,894,174 | 4.52 |
| **LG6** | 31 | 10 | 26,059,173 | 5.64 | 21,561,038 | 4.67 |
| **LG7** | 31 | 16 | 23,858,326 | 5.16 | 19,972,339 | 4.32 |
| **LG8** | 27 | 10 | 19,630,612 | 4.25 | 14,489,537 | 3.14 |
| **LG9** | 27 | 6 | 23,321,646 | 5.05 | 19,734,921 | 4.27 |
| **LG10** | 27 | 12 | 24,858,449 | 5.38 | 21,342,552 | 4.62 |
| **LG11** | 24 | 9 | 19,198,892 | 4.16 | 15,674,165 | 3.39 |
| **LG12** | 23 | 9 | 24,171,007 | 5.23 | 20,783,222 | 4.50 |
| **LG13** | 23 | 12 | 22,892,374 | 4.96 | 19,008,957 | 4.11 |
| **LG14** | 16 | 8 | 19,180,807 | 4.15 | 18,486,535 | 4.00 |
| **LG15** | 12 | 6 | 23,327,737 | 5.05 | 20,221,635 | 4.38 |
| **Total** | **461** | **196** | **373,442,800** | **80.83** | **309,412,257** | **66.97** |
| ***I. triloba*** |  |  |  |  |  |  |
| **LG1** | 21 | 13 | 38,073,535 | 8.32 | 38,039,483 | 8.31 |
| **LG2** | 6 | 3 | 27,163,338 | 5.93 | 27,138,914 | 5.93 |
| **LG3** | 13 | 8 | 33,436,119 | 7.30 | 33,406,022 | 7.30 |
| **LG4** | 19 | 9 | 36,325,649 | 7.93 | 36,266,339 | 7.92 |
| **LG5** | 14 | 10 | 32,557,749 | 7.11 | 32,540,226 | 7.11 |
| **LG6** | 18 | 9 | 27,621,998 | 6.03 | 27,583,017 | 6.02 |
| **LG7** | 15 | 12 | 28,880,297 | 6.31 | 28,766,353 | 6.28 |
| **LG8** | 14 | 10 | 20,894,540 | 4.56 | 20,824,755 | 4.55 |
| **LG9** | 12 | 8 | 31,779,738 | 6.94 | 31,759,800 | 6.94 |
| **LG10** | 22 | 13 | 29,788,665 | 6.51 | 29,636,812 | 6.47 |
| **LG11** | 13 | 10 | 25,628,617 | 5.60 | 25,587,827 | 5.59 |
| **LG12** | 12 | 6 | 28,329,316 | 6.19 | 27,972,185 | 6.11 |
| **LG13** | 13 | 11 | 32,054,333 | 7.00 | 32,046,261 | 7.00 |
| **LG14** | 9 | 6 | 23,643,743 | 5.16 | 23,630,883 | 5.16 |
| **LG15** | 15 | 8 | 27,097,266 | 5.92 | 27,077,160 | 5.91 |
| **Total** | **216** | **136** | **443,274,903** | **96.82** | **442,276,037** | **96.60** |

Percentage of anchored and oriented bases were calculated based on the assembly sizes of 461,997,559 bp (*I. trifida*) and 457,835,428 bp (*I. triloba*).

**Supplementary Table 4. Summary of the syntenic analysis between *Ipomoea* species (*I. trifida*, *I. triloba and I. nil*) and grape**

| **Species** | **Number of total gene models** | **Number of gene models in sytenic blocks** | | | |
| --- | --- | --- | --- | --- | --- |
|  |  | **(percentage of total gene models)** | | | |
|  |  | **Total** | ***Ipomoea*:grape=1:1** | ***Ipomoea*:grape=2:1** | ***Ipomoea*:grape=3:1** |
| *I. trifida* | 32,301 | 23,560 (72.9%) | - | - | - |
| Grape | 26,346 | 21,060 (79.9%) | 4,423 (16.8%) | 7,139 (27.1%) | 9,502 (36.1%) |
| *I. triloba* | 31,426 | 25,408 (80.9%) | - | - | - |
| Grape | 26,346 | 21,288 (80.8%) | 3,958 (15.0%) | 5,874 (22.3%) | 11,142 (42.29%) |
| *I. nil* | 42,783 | 28,989 (67.8%) | - | - | - |
| Grape | 26,346 | 21,455 (81.4%) | 4,634 (17.6%) | 6,520 (24.8%) | 9,754 (37.0%) |

**Supplementary Table 5. Variants detected in the Mwanga Diversity Panel**

|  | ***I. trifida*** | ***I. triloba*** |
| --- | --- | --- |
| Total variant loci | 6,090,896 | 6,660,594 |
| Multi-allelic loci | 306,839 | 359,205 |
| Total bi-allelic loci, no missing genotypes | 1,966,980 | 2,187,783 |
| SNPs | 1,787,193 | 1,994,091 |
| Insertions | 92,357 | 101,094 |
| Deletions | 87,093 | 92,210 |
| Combination | 337 | 388 |

**Supplementary References**

1. Davidson, R. M. *et al.* Utility of RNA sequencing for analysis of maize reproductive transcriptomes. *Plant Genome J.* **4,** 191 (2011).
2. Zhong, S. *et al.* High-throughput Illumina strand-specific RNA sequencing library preparation. *Cold Spring Harb. Protoc.* **2011,** 940–949 (2011).
3. Bolger, A. M., Lohse, M. & Usadel, B. Trimmomatic: a flexible trimmer for Illumina sequence data. *Bioinformatics* **30,** 2114–2120 (2014).
4. Morgan, M. *et al.* ShortRead: A bioconductor package for input, quality assessment and exploration of high-throughput sequence data. *Bioinformatics* **25,** 2607–2608 (2009).
5. Marcais, G., Yorke, J. A. & Zimin, A. QuorUM: An error corrector for Illumina reads. *PLoS One* **10,** e0130821 (2015).
6. Berlin, K. *et al.* Assembling large genomes with single-molecule sequencing and locality-sensitive hashing. *Nat. Biotechnol.* **33,** 623–630 (2015).
7. Martin, M. Cutadapt removes adapter sequences from high-throughput sequencing reads. *EMBnet. J.* **17,** 10–12 (2011).
8. Kim, D. *et al.* TopHat2: accurate alignment of transcriptomes in the presence of insertions, deletions and gene fusions. *Genome Biol.* **14,** R36 (2013).
9. Grabherr, M. G. *et al.* Full-length transcriptome assembly from RNA-Seq data without a reference genome. *Nat. Biotechnol.* **29,** 644–652 (2011).
10. Shelton, J. M. *et al.* Tools and pipelines for BioNano data: molecule assembly pipeline and FASTA super scaffolding tool. *BMC Genomics* **16,** 734 (2015).
11. Murray, M. G. & Thompson, W. F. Rapid isolation of high molecular weight plant DNA. *Nucleic Acids Res.* **8,** 4321–5 (1980).
12. Zhou, C. *et al.* Assembly of whole-chromosome pseudomolecules for polyploid plant genomes using outcrossed mapping populations. *bioRxiv* https://www.biorxiv.org/content/early/2017/03/22/119271 (2017).
13. Kielbasa, S. M., Wan, R., Sato, K., Horton, P. & Frith, M. C. Adaptive seeds tame genomic sequence comparison. *Genome Res.* **21,** 487–493 (2011).
14. Kim, D., Langmead, B. & Salzberg, S. L. HISAT: a fast spliced aligner with low memory requirements. *Nat. Methods* **12,** 357–360 (2015).
15. Anders, S. & Huber, W. Differential expression analysis for sequence count data. *Genome Biol.* **11,** R106 (2010).
16. Yang, J. *et al.* Haplotype-resolved sweet potato genome traces back its hexaploidization history. *Nat. Plants* **3,** 696–703 (2017).
17. Hoshino, A. *et al.* Genome sequence and analysis of the Japanese morning glory *Ipomoea nil*. *Nat. Commun.* **7,** 13295 (2016).
18. Si, Z. *et al.* A genome-wide BAC-end sequence survey provides first insights into sweetpotato (*Ipomoea batatas* (L.) Lam.) genome composition. *BMC Genomics* **17,** 945 (2016).
19. Simão, F. A., Waterhouse, R. M., Ioannidis, P., Kriventseva, E. V. & Zdobnov, E. M. BUSCO: assessing genome assembly and annotation completeness with single-copy orthologs. *Bioinformatics* **31,** 3210–3212 (2015).
20. David, M. C. *et al.* Gene pool subdivision of East African Sweetpotato parental material. *Crop Sci.* doi: 10.2135/cropsci2017.11.0695 (2018).
21. Dellaporta, S., Wood, J. & Hicks, J. Dellaporta DNA extraction: a plant DNA minipreparation: version II. *Plant Mol. Biol. Report.* **1,** 19–21 (1983).
22. Mace, E. S., Buhariwalla, K. K., Buhariwalla, H. K. & Crouch, J. H. A high-throughput DNA extraction protocol for tropical molecular breeding programs. *Plant Mol. Biol. Report.* **21,** 459–460 (2003).
23. Quinlan, A. R. & Hall, I. M. BEDTools: a flexible suite of utilities for comparing genomic features. *Bioinformatics* **26,** 841–2 (2010).
24. Braz, G. T. *et al.* Comparative Oligo-FISH Mapping: An efficient and powerful methodology to reveal karyotypic and chromosomal evolution. *Genetics* **208,** 513–523 (2018).
25. Wang, K., Li, M. & Hakonarson, H. ANNOVAR: functional annotation of genetic variants from high-throughput sequencing data. *Nucleic Acids Res.* **38,** e164 (2010).
26. Garrison, E. & Marth, G. Haplotype-based variant detection from short-read sequencing. (2012).
27. McKenna, A. *et al.* The genome analysis toolkit: a MapReduce framework for analyzing next-generation DNA sequencing data. *Genome Res.* **20,** 1297–1303 (2010).
28. Pham, G. M. *et al.* Extensive genome heterogeneity leads to preferential allele expression and copy number-dependent expression in cultivated potato. *Plant J.* **92,** 624–637 (2017).
29. *Amborella* Genome Project. The *Amborella* genome and the evolution of flowering plants. *Science* **342**, 1241089 (2013).
30. Doyle & JJ. A rapid DNA isolation procedure for small quantities of fresh leaf tissue. *Phytochem. Bull. Bot. Soc. Am.* **19,** 11–15 (1987).
31. Štorchová, H. *et al.* An improved method of DNA isolation from plants collected in the field and conserved in saturated NaCl/CTAB solution. *Taxon* **49,** 79 (2000).
32. Hirakawa, H. *et al.* Survey of genome sequences in a wild sweet potato, *Ipomoea trifida* (H. B. K.) G. Don. *DNA Res.* **22,** 171–179 (2015).
