# Supplementary material for: Genome sequences of two diploid wild relatives of cultivated sweetpotato reveal targets for genetic improvement
Source: Nat Commun. 2018 Nov 2;9:4580. doi: 10.1038/s41467-018-06983-8 (PMC6214957; doi:10.1038/s41467-018-06983-8)
Supplement: Supplementary file 3 — Description of Additional Supplementary Files [file 41467_2018_6983_MOESM3_ESM.pdf]

Description of Additional Supplementary Files

Supplementary Data 1
Description: Summary of RNA-Seq data

Supplementary Data 2
Description: Summary of repeats annotated using RepeatMasker and the custom repeat libraries

Supplementary Data 3
Description: Lineage-specific gene family expansion in *I. trifida* and *I. triloba*

Supplementary Data 4
Description: Gene ontology enrichment tests for various gene categories (highlighted significant *p*-values corrected by Bonferroni method)

Supplementary Data 5
Description: Expression levels of sporamin genes during storage root development

Supplementary Data 6
Description: Accessions of the Mwanga Diversity Panel

Supplementary Data 7
Description: Carotenoid-associated Arabidopsis genes and homologs in *I. trifida* and *I.triloba* as identified by BLASTP. Only the most significant isoform for each gene shown. Cut-offs of 70% coverage and 1e-40 e-value.

Supplementary Data 8
Description: Accession information for individuals included in phylogenetic analyses in this study
