# Supplementary material for: Genome sequences of two diploid wild relatives of cultivated sweetpotato reveal targets for genetic improvement
Source: Nat Commun. 2018 Nov 2;9:4580. doi: 10.1038/s41467-018-06983-8 (PMC6214957; doi:10.1038/s41467-018-06983-8)
Supplement: Supplementary file 12 — Reporting Summary [file 41467_2018_6983_MOESM12_ESM.pdf]

nature research

Corresponding author(s): Zhangjun Fei & C. Robin Buell

Reporting Summary

Nature Research wishes to improve the reproducibility of the work that we publish. This form provides structure for consistency and transparency in reporting. For further information on Nature Research policies, see Authors & Referees and the Editorial Policy Checklist.

Statistical parameters

When statistical analyses are reported, confirm that the following items are present in the relevant location (e.g. figure legend, table legend, main text, or Methods section).

n/a	Confirmed	
☐	☒	The exact sample size (*n*) for each experimental group/condition, given as a discrete number and unit of measurement
☐	☒	An indication of whether measurements were taken from distinct samples or whether the same sample was measured repeatedly
☐	☒	The statistical test(s) used AND whether they are one- or two-sided<br>*Only common tests should be described solely by name; describe more complex techniques in the Methods section.*
☐	☒	A description of all covariates tested
☐	☒	A description of any assumptions or corrections, such as tests of normality and adjustment for multiple comparisons
☐	☒	A full description of the statistics including central tendency (e.g. means) or other basic estimates (e.g. regression coefficient) AND variation (e.g. standard deviation) or associated estimates of uncertainty (e.g. confidence intervals)
☐	☒	For null hypothesis testing, the test statistic (e.g. *F*, *t*, *r*) with confidence intervals, effect sizes, degrees of freedom and *P* value noted<br>*Give P values as exact values whenever suitable.*
☒	☐	For Bayesian analysis, information on the choice of priors and Markov chain Monte Carlo settings
☒	☐	For hierarchical and complex designs, identification of the appropriate level for tests and full reporting of outcomes
☒	☐	Estimates of effect sizes (e.g. Cohen's *d*, Pearson's *r*), indicating how they were calculated
☐	☒	Clearly defined error bars<br>*State explicitly what error bars represent (e.g. SD, SE, CI)*

Our web collection on statistics for biologists may be useful.

Software and code

Policy information about availability of computer code

Data collection	No software was used for data collection.
Data analysis	No commercial and custom code was used in this study. We only used freely available bioinformatics software our data analysis.

For manuscripts utilizing custom algorithms or software that are central to the research but not yet described in published literature, software must be made available to editors/reviewers upon request. We strongly encourage code deposition in a community repository (e.g. GitHub). See the Nature Research guidelines for submitting code & software for further information.

Data

Policy information about availability of data

All manuscripts must include a data availability statement. This statement should provide the following information, where applicable:

- Accession codes, unique identifiers, or web links for publicly available datasets
- A list of figures that have associated raw data
- A description of any restrictions on data availability

The genome assemblies and raw genome reads of I. trifida and I. triloba have been deposited into GenBank BioProject under accessions PRJNA428214 (https://www.ncbi.nlm.nih.gov/bioproject/PRJNA428214/) and PRJNA428241 (https://www.ncbi.nlm.nih.gov/bioproject/PRJNA428241/), respectively. Raw 10x Genomics, genome resequencing and RNA-Seq reads are available in the National Center for Biotechnology Information Sequence Read Archive under accessions SRP161954

(https://www.ncbi.nlm.nih.gov/sra/?term=SRP161954), SRP162006 (https://www.ncbi.nlm.nih.gov/sra/?term=SRP162006), SRP132113 (https://www.ncbi.nlm.nih.gov/sra/?term=SRP132113), SRP132112 (https://www.ncbi.nlm.nih.gov/sra/?term=SRP132112), SRP162112 (https://www.ncbi.nlm.nih.gov/sra/?term=SRP162112), SRP162110 (https://www.ncbi.nlm.nih.gov/sra/?term=SRP162110), and SRP162021 (https://www.ncbi.nlm.nih.gov/sra/?term=SRP162021). The pseudomolecules, genome annotation, expression abundances, BLAST server, and Jbrowse instance are available in the Sweetpotato Genomics Resource (http://sweetpotato.plantbiology.msu.edu). In addition, the genome assemblies, annotated genes, expression abundances, and variants of the MDP are available via the Dryad Digital Repository (to be made public upon acceptance; for review purposes the files can be accessed at this temporary URL: https://datadryad.org//review?doi=doi:10.5061/dryad.b9m61cg).

Field-specific reporting

Please select the best fit for your research. If you are not sure, read the appropriate sections before making your selection.

☒ Life sciences ☐ Behavioural & social sciences ☐ Ecological, evolutionary & environmental sciences

For a reference copy of the document with all sections, see nature.com/authors/policies/ReportingSummary-flat.pdf

Life sciences study design

All studies must disclose on these points even when the disclosure is negative.

Sample size	For RNA-Seq experiment of sweetpotato root development, we used four biological replicates. The number of replicates (3 or 4) are commonly used in RNA-Seq studies.
Data exclusions	For genome and RNA-Seq data, we only excluded sequences that were of low quality and potential contaminants from the analysis. This is standard for these types of analyses. SNP data were also filtered according to commonly used standards. All these were described in detail in the manuscript.
Replication	We used various approaches to evaluate our genome assemblies, confirming the assemblies are of high-quality.
Randomization	This is not relevant to our study since we are reporting genome assemblies, comparative genomic analyses, and expression profiling analysis of plant species.
Blinding	Blinding was not relevant to our study since our study is on genome and transcriptome analysis of plant species.

Reporting for specific materials, systems and methods

Materials & experimental systems

n/a	Involved in the study	
☒	☐	Unique biological materials
☒	☐	Antibodies
☒	☐	Eukaryotic cell lines
☒	☐	Palaeontology
☒	☐	Animals and other organisms
☒	☐	Human research participants

Methods

n/a	Involved in the study	
☒	☐	ChIP-seq
☒	☐	Flow cytometry
☒	☐	MRI-based neuroimaging
